# A Case Demonstrating the Relationship Between Intraocular Pressure and Blood Pressure Through Personal Health Monitoring

**DOI:** 10.7759/cureus.92884

**Published:** 2025-09-21

**Authors:** Masaki Tanito

**Affiliations:** 1 Department of Ophthalmology, Shimane University Faculty of Medicine, Izumo, JPN

**Keywords:** blood pressure, glaucoma, home tonometry, intraocular pressure, n-of-1 trial, personalized medicine, time series analysis

## Abstract

Glaucoma is increasingly recognized as a multifactorial disease influenced by both ocular and systemic factors. Among systemic parameters, blood pressure (BP) has been reported to affect intraocular pressure (IOP), although the relationship is complex and may vary among individuals. This report describes the case of a 51-year-old Japanese male physician without glaucoma who voluntarily performed self-monitoring of IOP, BP, and body weight (BW) over a 24-month period using home tonometry, a wearable sphygmomanometer, and a digital scale. A total of 1,243 right-eye and 1,240 left-eye IOP measurements, 1,210 BP recordings, and 629 BW records were collected. Time series analysis using an AutoRegressive Integrated Moving Average with eXogenous variables (ARIMAX) model, configured as SARIMAX(0,1,1), revealed that systolic BP (SBP) was significantly and positively associated with IOP (IOP changed by 0.27 mmHg for every 10 mmHg change in SBP), whereas diastolic BP (DBP) and BW showed no significant associations. Right- and left-eye IOP fluctuations occurred in phase, suggesting the influence of systemic or environmental factors beyond local ocular determinants. This case illustrates the potential utility of N = 1 analyses and personalized health monitoring in elucidating the role of systemic factors in IOP regulation and highlights future possibilities for incorporating continuous systemic and ocular measurements into individualized glaucoma management strategies.

## Introduction

Glaucoma is increasingly recognized as a multifactorial disease, influenced not only by ocular factors but also by a variety of systemic conditions that may modify its onset and progression [1]. Several large-scale epidemiological studies have reported a positive correlation between blood pressure (BP) and intraocular pressure (IOP) [2-4]. Conversely, it has also been shown that the prevalence of glaucoma increases in individuals with either low or high BP [5]. These findings suggest that the effects of BP on glaucoma are complex and may differ on a case-by-case basis.

In recent years, interest in personal health monitoring has grown, with particular attention given to devices such as wearable BP monitors [5] and home tonometry systems [6]. In the present report, the case voluntarily performed self-monitoring of IOP, BP, and body weight (BW) over a period of two years and provided these data for clinical evaluation. This report details an N = 1 observational study of the association between IOP and systemic parameters, specifically BP and BW, based on this unique dataset. Such an attempt may be considered important in demonstrating the practice of personal health monitoring.

## Case presentation

A 51-year-old Japanese male physician presented for a glaucoma screening examination. His medical history included hypertension and dyslipidemia, for which he was receiving bisoprolol fumarate (2.5 mg/day, in the morning) and lovastatin calcium (5 mg/day, in the morning). There were no other notable ocular or systemic medical histories.

**Figure 1:**
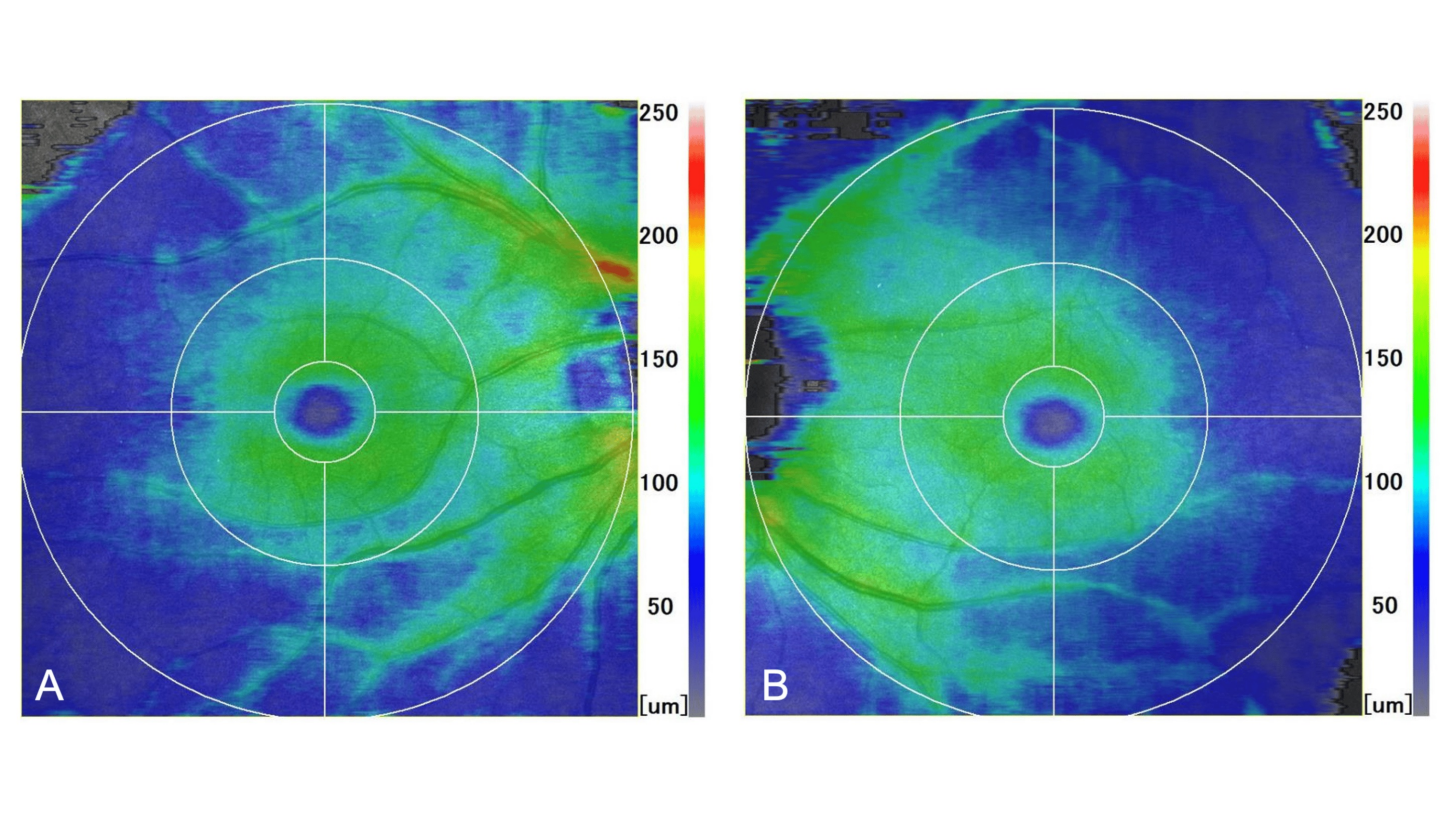
Macular optical coherence tomography (OCT) scans of the right (A) and left (B) eyes No apparent glaucomatous nerve fiber layer defect is observed.

Best-corrected visual acuity was 1.0 in both eyes, with high myopia of -8.25 D in the right eye and -7.5 D in the left eye. Goldmann applanation tonometry revealed an IOP of 18 mmHg in the right eye and 16 mmHg in the left eye. The anterior segment was unremarkable, and gonioscopy confirmed open angles. The vertical cup-to-disc ratio was 0.8 bilaterally. The central corneal thickness measured by specular microscopy was 556 μm in the right eye and 530 μm in the left eye (EM-3000, Tomey Corporation, Nagoya, Japan). The axial length was 28.75 mm in the right eye and 27.56 mm in the left eye (OA-2000, Tomey Corporation). Optical coherence tomography (RS-3000 Advance 2, NIDEK CO., LTD., Gamagori, Japan) demonstrated no definite glaucomatous changes in the macula or optic nerve head (Figures 1A-1B). Humphrey visual field testing (Central 30-2; Carl Zeiss Meditec, Dublin, CA) showed mean deviation values of -1.73 dB in the right eye and -2.79 dB in the left eye. Although mild sensitivity reduction was observed around the optic disc, consistent with myopic conus, there was no glaucomatous visual field loss. Based on these findings, glaucoma was considered unlikely.

**Figure 2:**
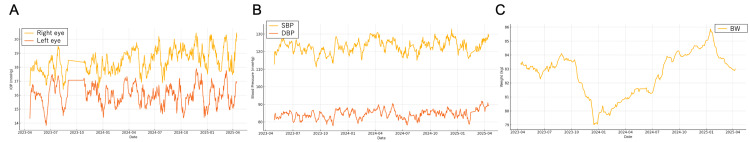
Longitudinal trends of IOP (A), BP (B), and BW (C) throughout the observation period All data are presented as 10-point moving averages. BW, body weight; DBP, diastolic blood pressure; IOP, intraocular pressure; SBP, systolic blood pressure

Because the IOP was slightly high, the patient subsequently purchased an iCare HOME2 (iCare Finland Oy, Vantaa, Finland) device and performed self-monitoring of IOP over a 24-month period. At the same time, he also performed self-measurement of BP using a wristwatch-type sphygmomanometer (HCR-6900T, OMRON Healthcare Co., Ltd., Kyoto, Japan) and BW using a digital scale (KRD-703T, OMRON Healthcare Co., Ltd.). IOP was measured nearly every morning upon waking, with additional measurements taken at arbitrary times. BP was also measured primarily each morning, with additional readings obtained during the day. BW was recorded almost every evening before dinner. There were no changes in oral medications during the course of follow-up. Over the 24 months, measurement counts were as follows: 1,243 readings of right eye IOP, 1,240 readings of left eye IOP, 1,210 BP readings, and 629 BW measurements. The longitudinal trends of these variables are presented in Figures 2A-2C. The average values were: right eye IOP, 18.3 mmHg; left eye IOP, 16.0 mmHg; systolic BP (SBP), 122.9 mmHg; diastolic BP (DBP), 84.3 mmHg; and BW, 82.6 kg.

To evaluate the influence of BP and BW on IOP, time series analysis has been conducted using an AutoRegressive Integrated Moving Average with eXogenous variables (ARIMAX) model, configured as SARIMAX(0,1,1) with SBP, DBP, and BW as exogenous variables. Model fitting was performed in Python using the statsmodels library, specifically the class statsmodels.tsa.statespace.sarimax.SARIMAX. To justify the use of first-order differencing in this model, we evaluated the stationarity of the IOP time series using both the augmented Dickey-Fuller (ADF) and Kwiatkowski-Phillips-Schmidt-Shin (KPSS) tests. The ADF test rejected the null hypothesis of a unit root (p < 0.001), while the KPSS test suggested non-stationarity (p = 0.010). This combination is characteristic of trend-stationary series and supported the use of d = 1. The exogenous variables (SBP, DBP, and BW) were used in their level form. Although this specification implies that BP levels predict changes in IOP, we consider this structure physiologically plausible in the context of personal health monitoring. This framework provides a flexible state space approach for estimating ARIMA-type models with optional seasonality and exogenous regressors via maximum likelihood estimation. For model construction, when multiple readings were obtained on the same day, the daily average was used, and the mean of the right and left eye IOP was adopted for that day. In this case, no IOP-lowering treatment was administered during the observation period. As illustrated in Figure 2, the time-series patterns of IOP in the right and left eyes were concordant, justifying the use of their average for daily analysis in this case.

The analysis demonstrated that SBP was significantly and positively associated with IOP (coefficient = 0.027, p = 0.010), indicating that increases in SBP corresponded to modest rises in IOP (Table 1). In contrast, the associations of DBP (coefficient = 0.020, p = 0.127) and BW (coefficient = 0.075, p = 0.219) with IOP were not statistically significant (Table 1). The model included a moving average component of order 1 (MA(1)), which was highly significant (p < 0.001), suggesting that short-term prediction errors contributed substantially to IOP fluctuations. The residual variance (σ²) was estimated at 3.05, indicating a reasonable level of prediction error (Table 1). Diagnostic testing confirmed that the residuals followed a normal distribution (Jarque-Bera p = 0.93) and demonstrated no significant autocorrelation (Ljung-Box p = 0.68), supporting the adequacy of the model fit.

**Table 1:** Results of time series analysis of the effects of BP and BW on IOP fluctuations BW: body weight; DBP: diastolic blood pressure; MA (1): moving average of order 1; SBP: systolic blood pressure; σ²: variance of the model residuals

Parameters	Regression coefficient	p-value
SBP	+0.027	0.010
DBP	+0.020	0.127
BW	+0.075	0.219
MA (1)	-0.988	<0.001
σ²	3.049	-

## Discussion

Several time series models, including ARIMAX and seasonal ARIMAX, were tested to investigate the association between IOP and systemic variables. Among these, the ARIMAX(0,1,1) model incorporating three exogenous variables provided the most interpretable and stable results as indicated by the lowest Akaike Information Criterion value. Previous analyses using health checkup data, mostly from non-glaucomatous individuals, have reported that only SBP was associated with IOP [7], while other studies have reported associations of both SBP and DBP with IOP [8]. These findings suggest that the impact of BP on IOP may vary among individuals. In this particular case, continuous self-monitoring demonstrated that SBP was significantly correlated with IOP. The patient was undergoing antihypertensive treatment with a β-blocker, and BP was well controlled. If this patient were to develop glaucoma with poorly controlled BP, achieving proper BP control could potentially lead to IOP reduction, providing a strategic component of glaucoma management. However, the observed effect of BP on IOP in this case was significant but marginal, and it remains unclear whether further BP control would actually reduce IOP. Moreover, this patient did not have glaucoma, and the study is limited to a single case (N = 1). Therefore, caution is warranted when generalizing the findings. This case report is intended primarily to highlight a potential topic for future research.

Interestingly, the fluctuations in right- and left-eye IOP occurred in phase (Figure 2A). The synchronized variation of IOP between both eyes cannot be explained solely by local ocular factors acting independently. This dataset suggests that extraocular systemic factors or environmental influences, such as body physiology or ambient temperature, play a critical role in IOP regulation. Positive correlations between BW or body mass index and IOP have been reported in both glaucomatous and non-glaucomatous eyes [9-11]. In this case, BW fluctuated by as much as 9 kg over the two-year observation period, but no significant association with IOP was detected. This finding further emphasizes that the effects of systemic factors on IOP may differ across individuals.

In this case, IOP was measured in the morning, BP during the day, and BW in the evening using commercially available home-use devices; while these devices are widely used in self-monitoring, factors such as posture and device positioning (e.g., probe angle for tonometry, cuff height for BP) may introduce variability, and timing mismatches across parameters should be considered in interpreting the results. While the present study focused on the effects of BP and BW on IOP, various other factors - including diet, sleep, exercise, and environmental temperature - are also thought to influence IOP [1]. The development of monitoring techniques for such factors will likely contribute to a better understanding of the relationship between lifestyle and glaucoma. Furthermore, this study is based solely on the observations of a single non-glaucoma case, and caution is warranted when generalizing the results. In particular, there is no evidence to suggest that BP control is beneficial for IOP management in glaucoma patients; therefore, the findings should be interpreted with caution.

In line with the CARE guidelines, the case’s perspective was also considered. The case noted that automated systems for daily measurements would be desirable and that it would be beneficial if the relationships between IOP and lifestyle factors, such as BP, BW, step count, sleep duration, and caffeine or alcohol intake, could be visualized at the individual level. The case further emphasized that the advancement of simple yet highly accurate monitoring devices will be essential for the practical implementation of personal health monitoring. N-of-1 trials are considered a valuable methodology for the practice of personalized medicine [12]. This case further illustrates how personalized health monitoring, through high-frequency and home-based measurements, may complement conventional clinic-based or cross-sectional studies by capturing individual-specific physiological fluctuations over time, an approach that aligns with the principles of personalized medicine. 

## Conclusions

This case serves as a notable example of the potential utility of personalized health monitoring. With the future establishment of devices enabling continuous BP and IOP measurement, together with robust analytical methods, it may become increasingly feasible to incorporate systemic factors into the practical management of glaucoma. It should be noted, however, that there is currently no evidence that BP control lowers IOP in glaucoma patients, and clinical benefit cannot yet be concluded from this case.
